# Congenital Midline Cervical Skin Bridge

**Published:** 2011-07-30

**Authors:** Bilal Mirza, Afzal Sheikh

**Affiliations:** Department of Pediatric Surgery, The Children's Hospital and the Institute of Child Health Lahore, Pakistan

 A 6-month-old male infant presented in outpatient department with a congenital skin bridge in midline of the neck region. The bridge was causing difficulty in neck movements. On clinical examination the infant was healthy with a 1x4 cm skin bridge extending from the cervical region at the level of hyoid bone to the suprasternal notch, in the midline (Fig. 1, 2). There were no other problems. The skin bridge was excised by making elliptical incisions on either sides of the bridge. The skin beneath the excised skin bridge was slightly thick as compared to the surrounding area (Fig. 3). The thickened skin became normal at 2 months follow-up. Histopathology of the tissue revealed as normal skin and subcutaneous tissue.

**Figure:**
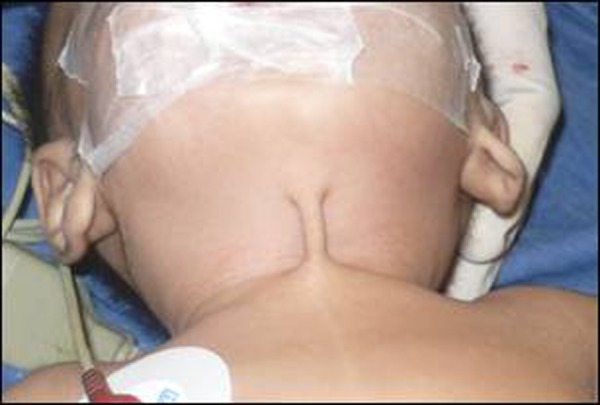
Figure 1: Anterior view

**Figure:**
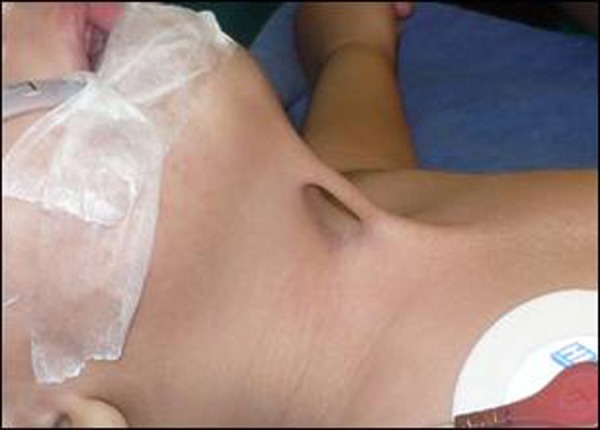
Figure 2: Lateral view

**Figure:**
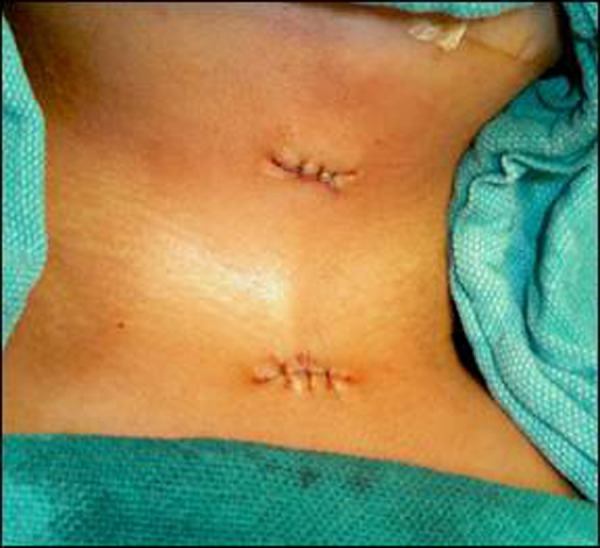
Figure 3: After excision, the skin in the midline was slightly thick in comparison to the surrounding skin

## DISCUSSION

Congenital midline cervical skin bridge is an extremely rare entity. It is a benign lesion composed mainly of skin. No major structures like arteries veins or nerves passes through it [1]. Its blood supply can be speculated to come from the skin of suprasternal notch as while excision a small subcutaneous vessel was secured with the suture.

Only one case has been reported in 2008 by Kawar et al. The etiology of congenital midline cervical skin bridge, as suggested by Kawar et al, is failure of resorption of skin and subcutaneous tissue at the time of fusion of neck tissues in the midline or epithelialization of an amniotic band [1]. We believe that occurrence of this anomaly is not a chance fusion of skin as in amniotic bands but results due to specific underlying phenomenon like many other congenital anomalies.

Restriction of neck extension and cosmetic problems are the main indications of the surgery. The case of Kawar et al presented at 3 months age whereas our patient presented at 6 months age. We believe that on account of delayed presentation the skin beneath the bridge became slightly thickened due to failure of neck extension (natural tendency of tissues). After excision of the bridge the skin became normal in texture.

## Footnotes

**Source of Support:** Nil

**Conflict of Interest:** None declared
